# Natural Sources of Therapeutic Agents Used in Skin Conditions

**DOI:** 10.3390/life14040492

**Published:** 2024-04-10

**Authors:** Monica Dinu, Alin Laurențiu Tatu, Dorin Ioan Cocoș, Lawrence Chukwudi Nwabudike, Ana Maria Chirilov, Claudia Simona Stefan, Kamel Earar, Olimpia Dumitriu Buzia

**Affiliations:** 1 Centre in the Medical-Pharmaceutical Field, Faculty of Medicine and Pharmacy, “Dunarea de Jos” University of Galati, 800008 Galati, Romania; monica.dinu24@gmail.com (M.D.); claudia.stefan@ugal.ro (C.S.S.); erar_dr.kamel@yahoo.com (K.E.); buzia_olimpia@yahoo.com (O.D.B.); 2 Clinical Medical Department, Faculty of Medicine and Pharmacy, “Dunarea de Jos” University of Galati, 800008 Galati, Romania; dralin_tatu@yahoo.com; 3 Dermatology Department, “Sf. Cuvioasa Parascheva” Clinical Hospital of Infectious Diseases, 800179 Galati, Romania; 4 Multidisciplinary Integrative Center for Dermatologic Interface Research MIC-DIR, 800010 Galati, Romania; 5 Nicolae Paulescu Institute, 030167 Bucharest, Romania; chukwudi.nwabudike@live.com

**Keywords:** bee venom, *Ficus carica*, *Geranium* essential oil, atopic dermatitis, acne, psoriasis, wounds, alopecia

## Abstract

Skin conditions are numerous and often have a major impact on patients’ quality of life, and effective and safe treatment is very important. The conventional drugs used for skin diseases are usually corticosteroids and antimicrobial products that can induce various side effects, especially with long-term use, which is why researchers are studying alternatives, especially biologically active natural products. Three products caught our attention: bee venom (BV), due to reported experimental results showing anti-inflammatory, antibacterial, antiviral, antioxidant, antimycotic, and anticancer effects, *Ficus carica* (FC) due to its demonstrated antioxidant, antibacterial, and anti-inflammatory action, and finally *Geranium* essential oil (GEO), with proven antifungal, antibacterial, anti-inflammatory, and antioxidant effects. Following a review of the literature, we produced this paper, which presents a review of the potential therapeutic applications of the three products in combating various skin conditions and for skin care, because BV, FC, and GEO have common pharmacological actions (anti-inflammatory, antibacterial, and antioxidant). We also focused on studying the safety of the topical use of BV, FC, and GEO, and new approaches to this. This paper presents the use of these natural therapeutic agents to treat patients with conditions such as vitiligo, melasma, and melanoma, as well as their use in treating dermatological conditions in patients with diabetes.

## 1. Introduction

The skin is the largest organ of the body; it consists of three distinct layers, represented by the epidermis, dermis, and subcutaneous adipose tissue, inside which are blood vessels, nerves, and appendages (hair follicles, sebaceous glands, sweat glands, and nails) [1]. For the human body, the skin acts as a barrier, providing protection against allergens, chemicals, toxins, and pathogens (fungi, bacteria, viruses, and parasites). It helps to regulate body temperature, as well as the amount of water and electrolytes [2]. When the dynamics that allow the skin to perform its multiple and complex functions are disrupted, various skin conditions can occur, which can sometimes have a significant physical, social, or psychological impact on patients, negatively affecting their quality of life [3].

Skin diseases are numerous, occur frequently, affect all ages, and can be classified into several common categories: skin rashes (acne, dermatitis, eczema, hives, psoriasis), viral infections (herpes simplex, herpes zoster, warts), bacterial infections (folliculitis, cellulitis, Lyme disease), pigmentation disorders (vitiligo, melasma), trauma (strokes, burns, cuts), fungal infections, parasitic infections, tumors, and cancers [4].

### Skin Conditions That Can Be Treated with Therapeutic Agents from Natural Sources

Atopic dermatitis is a chronic and relapsing inflammatory condition of the skin, caused by genetic, inflammatory, and immunological abnormalities, with a high incidence in recent years [5]. It is characterized by recurrent eczema, pruritus, and xerosis, and treatment options are limited, although antihistamines, topical corticosteroids, nonsteroidal anti-inflammatory drugs, and immunosuppressants are used [6,7,8]. These drugs can cause serious local and systemic adverse effects, such as nephrotoxicity, neurotoxicity, infections, and skin cancers [9,10,11]. 

Acne is a chronic dermatological disorder that is usually present on the face, arms, chest, or back, and mainly affects adolescents. It is the eighth most prevalent disease globally and affects approximately 10% of the world’s population [12]. It is pathologically characterized by the increased secretion of sebum, bacterial colonization of the sebaceous ducts, ductal cornification, inflammation, and the appearance of papules, pustules and nodules [13]. To kill the involved bacteria and combat inflammation, antibiotics are commonly used, but their frequent use carries the risk of resistant bacterial strains developing. Therefore, there is a major global interest in discovering alternative treatments for this condition that do not present side effects [14,15].

Psoriasis has been declared the fifth most important chronic non-contagious disease by the World Health Organization; it is a genetically determined, chronic, inflammatory skin condition that is characterized by erythematous plaques covered with silvery scales, especially on the extensor surfaces, scalp, and lumbosacral region [16,17].

Wound healing is a dynamic and complex process involving the replacement of affected tissue with living tissue, which consists of several overlapping phases: hemostasis with clot formation, inflammation, proliferation consisting of extracellular matrix biosynthesis, epithelialization, and angiogenesis, and the last phase, represented by tissue remodeling [18,19].

Alopecia (hair loss) is a condition with a negative impact on quality of life; it is especially associated with genetic causes, stress, or inflammation of the scalp. At present various topical, oral, or transplant operations are used as a treatment, but there is a desire to discover new therapeutic approaches with better results [20].

Wrinkles are a change in skin appearance induced by ultraviolet rays and the natural aging process, factors that induce decreased collagen production and reduced skin flexibility [21].

Melanoma is a form of skin cancer with increasing incidence and mortality globally, which is highly resistant to conventional chemotherapy drugs, which is why researchers are studying several natural products, including BV, for their potential anticancer effects [22].

Melasma is a skin condition characterized by the chronic overproduction of melanin, which manifests in the appearance of brown, irregular macules of varying intensity. These macules are located symmetrically on sun-exposed surfaces of the body, especially on the face, including the cheeks, nose, forehead, upper part of the eyebrows and lips, and on the chin [23]. Women are mainly affected (about 90% of cases), with a higher frequency among people with darker skin. The frequency of melasma varies between 1.5% and 33.3%, depending on the population, and during pregnancy, it can affect 50–70% of women [24]. Several factors can contribute to the development of melasma, including genetic predisposition, prolonged sun exposure, birth control pills, hormone replacement therapy, certain medications, or cosmetic use [24,25]. Melasma is a difficult-to-treat skin condition for which there is still no generally recognized remedy. Conventional treatments, including topical and oral treatments, along with skin restoration procedures such as chemical peels or light and laser treatments, provide temporary and uncertain results [25].

Morphea, also called localized scleroderma, is a rare inflammatory disease of the skin and subcutaneous tissue that affects adults and children equally and presents with an as-yet-unknown etiology. It is manifested by inflammation and fibrosis of the underlying skin and soft tissues, sometimes even the muscles, central nervous system, or bones, and to date, there is no specific cure, only immunosuppressants, intralesional, topical, oral steroids, and phototherapy [26]. 

Vitiligo is an autoimmune disease characterized by depigmentation of the skin and hair. Melanocytes are the specific cells responsible for skin pigmentation. Skin repigmentation can take a long time, involving melanocyte proliferation, melanogenesis, migration, or increased dendricity [27,28].

Fungal infections of the skin are clinically manifested by desquamation, erythema, pruritus, maceration, and sometimes painful lesions. They are contagious, unsightly, can affect anyone, and are caused by dermatophyte species, *Candida* or *Malassezia* [29].

Skin diseases are a global concern. Conventional treatments are limited by their side effects, so there is a desire to identify new molecules that are effective and safe to combat these conditions. Natural sources for biologically active agents can include plants and insects [30]. BV, FC, and GEO have aroused our interest due to their reported results, which include pharmacological actions (some common) that are potentially useful in treating some skin diseases.

## 2. Bee Venom

BV is a defense tool of the hive against predators and is secreted by a gland located in the abdominal cavity of bees *(Apis mellifera*) [31,32]. For thousands of years, people have used bee products in various forms to treat diseases, with the curative effects being mentioned in the Holy Quran, the Bible, and the Veda [33]. BV was used medically in ancient Egypt and Greece, and in China, BV therapy has been known and applied for about 5000 years [33]. Historically, BV has generally been associated with the treatment of inflammatory conditions, rheumatism, and skin diseases, as well as malaria [34,35]. The ancient Greek doctor Hippocrates (460-370 i.Hr) used BV as a treatment for baldness, and Ivan the Terrible (XV century) used it to cure gout [36,37]. Interest in this biological product continued, and in 1868, the Russians Lokumsky and Lubarsky published a paper titled “Bee Venom, a Remedy” [38].

Studies have shown that BV is a complex of peptides, enzymes, amines, lipids, carbohydrates, free amino acids, and minerals, as well as various volatile compounds [39]. Peptides such as melittin, apamine, sekapine, adolapin, tertiapine, the mast cell degranulation peptide, and prokamine are the main components of BV [40]. An important category in BV composition is proteins that act as enzymes, such as phospholipase A2, phospholipase B, acid phosphomonoesterase, hyaluronidase, phosphatase, and lysophospholipase, and, of these, phospholipase A2 is the main allergen in BV and has effects on inflammation and pain [41]. BV composition also includes biologically active amines represented by histamine, epinephrine, dopamine, and norepinephrine, about 20 volatile components, sugars (glucose and fructose), amino acids (y-aminobutyric acid and B-aminoisobutyric acid), and minerals (calcium, phosphorus and magnesium) [42,43]. Some studies have also reported the presence of toxic metals such as As, Ba, Cd, Sb, Pb, and Cr; therefore, when venom is used in pharmaceutical practice, it is important to identify metal contamination to ensure its quality and safety [44]. BV may have a different composition depending on the season, floral species, age of bees, and geographical location, with the composition possibly influencing the level of melittin, apamine, hyaluronidase, or phospholipase A2 [45,46].

**Figure 1:**
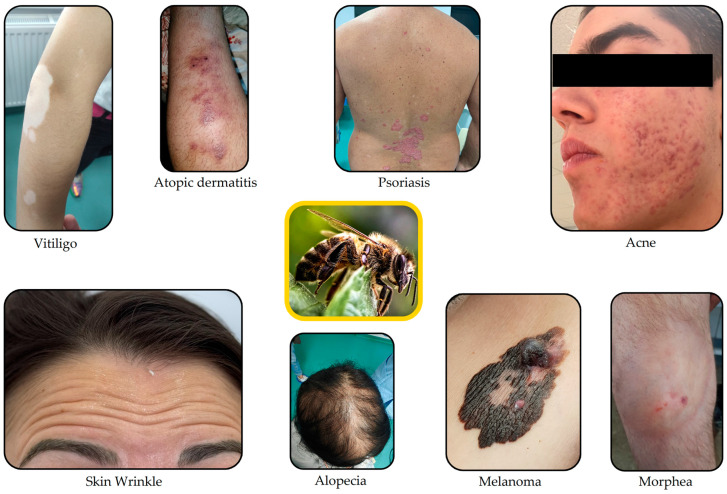
Skin diseases in which the therapeutic application of BV has been studied.

Numerous studies have demonstrated that BV is particularly rich in bioactive compounds that provide it with a variety of pharmacological activities, such as the following: anti-inflammatory, neuroprotective, analgesic, antioxidant, antibacterial, antiviral, antifungal, antidiabetic, anticancer, and antiatherosclerotic activities, as well as the ability to reduce the adverse effects of drugs already used in pharmaceutical practice [47,48,49,50,51,52,53,54,55,56,57,58,59]. As a novelty, due to its anti-inflammatory, antiviral, and immunomodulatory effects, BV has shown that it could be a potential complementary therapy against SARS-CoV-2 [60,61,62]. Research on animals demonstrated that the topical application of BV posed no risk of local adverse reactions, so it could be a potential means of caring for human skin as well [63]. Used in various studies, BV has been proven to have beneficial effects on the skin in acne, psoriasis, vitiligo, topical dermatitis, melanoma, morphea, wound treatment, and alopecia, as well as anti-aging effects (Figure 1) [64,65,66,67].

## 3. *Ficus carica*

The fig tree (*Ficus carica*) is a small tree that is considered to be part of the largest genera of angiosperm plants, part of the large family *Moraceae*, genus *Ficus,* order *Urticales*, and grows in temperate and tropical regions [68]. It is a heterozygotte and bears the popular name of “fig tree”, because it has small, greenish, pedunculate, fleshy flowers, which are not visible from the outside but closed in the receptacle and visible only to the fruit. All female flowers are self-pollinating (typical fig tree phenomenon) [68,69]. The fig tree has been used since ancient times due to its therapeutic properties in treating various dermatological conditions. Fourteenth-century documents attest to knowledge of the healing properties of the fig tree, inspired by the research of Dioscorides and Galenus of Pergamon [68,69,70].

**Figure 2:**
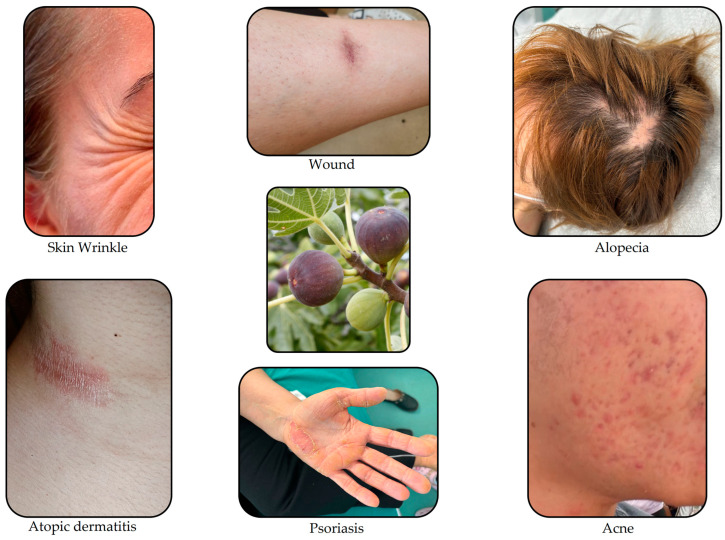
Skin diseases in which the therapeutic application of FC has been studied.

Traditional and advanced methods of investigation have allowed for the determination of about 126 chemical compounds in FC, including hydroxybenzoic acids, hydroxycinnamic acids, flavonoids, coumarins, furanocoumarins, volatile constituents, triterpenoids, and other substances, but the aromatic character and quality of the leaves are influenced by the volatile compounds they contain [70]. The variety of chemical constituents gives it various pharmacological actions, such as antioxidant, anticancer, hepatoprotective, hypoglycemic, antibacterial, antipyretic, anthelmintic, and anti-inflammatory properties, and due to its antioxidant and anti-inflammatory effects, fig extracts can play a significant role in skin care and maintaining skin health. Used in various studies, FC has been proven to have beneficial effects in various skin diseases (Figure 2) [70,71].

## 4. *Geranium* Essential Oil 

**Figure 3:**
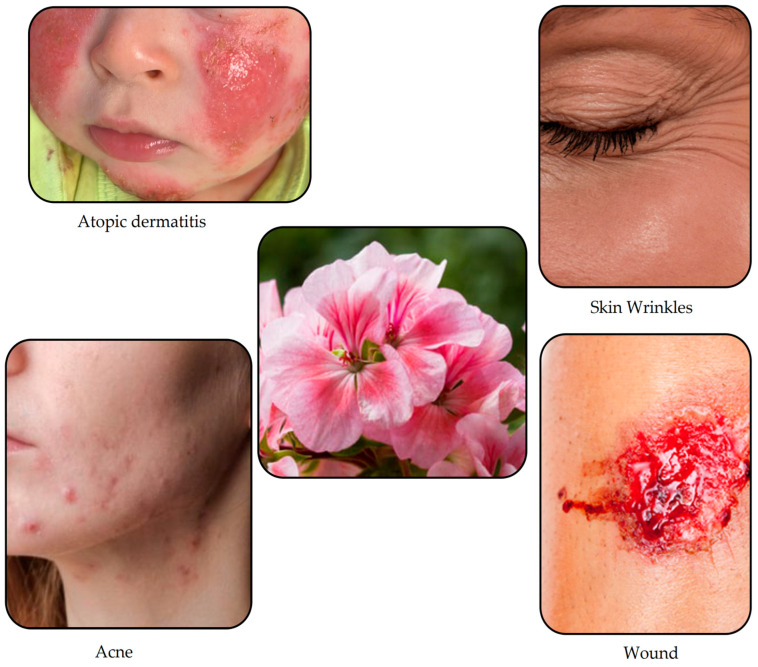
Skin diseases in which the therapeutic application of GEO has been studied.

South Africa is the origin of *Geranium*, which has over 300 species, and the family to which it belongs is *Geraniaceae*. The study focuses on *Pelargonium roseum* and *Pelargonium graveolens* species [72]. Current pharmacological and medical studies have shown that GEO can have broad-spectrum actions in the field of dermatology (Figure 3), with the following pharmacological actions: antiviral, anti-inflammatory, antimicrobial, oral cavity fresheners, astringent, healing, antifungal, tissue regeneration, pain relievers, antioxidants, and antitumor actions. These actions have a significant impact on the patient’s quality of life [73].

GEO can be used in dermatology due to its increased potential in cleansing, odorizing, and purifying the skin, with multiple benefits for the treatment of acne and other dermatological conditions of microbial origin. When the GEO’s destination is dermatological use, it is recommended to dilute it in a potential carrier oil (coconut oil or jojoba oil); for its use without dilution and in contact with skin is contraindicated [74].

**Figure 4:**
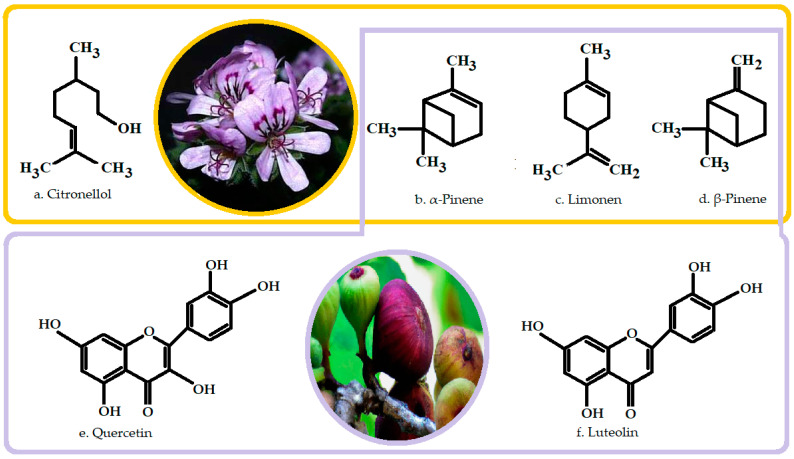
The chemical structure of the most important constituents of GEO (**a**–**d**) and FC (**b**–**f**). Common constituents (**b**–**d**).

Following the examination by gas chromatography, increased concentrations of citronellol of major importance were found in GEO, and smaller quantities of α-pinene, limonene and β-pinene, and phytoconstituents were also present in FC, with high-level antibacterial and antifungal actions (Figure 4) [5,73].

## 5. Scientific Evidence of the Beneficial Effects of Bee Venom, *Ficus carica,* and *Geranium* Essential Oil for Skin Care and Treatment

The desire to discover alternative therapeutic agents that can be successfully used in skin conditions without adverse reactions has led to the study of various natural substances, including BV, FC, and GEO.

### 5.1. Atopic Dermatitis

#### 5.1.1. Bee Venom in Atopic Dermatitis

An et al. used TNF-α/IFN-γ-stimulated human keratinocytes and demonstrated that BV and melittin suppressed the increased expression of chemokines such as CCL17 and CCL22, and proinflammatory cytokines including IL-6, IL-1β, and IFN-γ, by blocking NF-Kb and STAT signaling pathways. Furthermore, they studied the effects of BV and melittin in mouse models of female mice with 1-chloro-2-induced atopic dermatitis, 4-dinitrobenzene, treated with BV, melittin, or placebo, which were applied topically to shaved back skin five times weekly for 4 weeks. BV and melittin were found to restore abnormal epidermal differentiation by recovering filagrine expression and significantly alleviating symptoms of atopic dermatitis. These results indicate that bee venom and melittin may be therapeutically effective in treating this skin condition [75].

The pharmaceutical actions of melittin, the main component of BV, have been intensively studied. Kim et al. used patches of ovalbumin (OVA), an important protein in egg white, to induce in similar symptoms to atopic dermatitis in mouse models, such as thickening of the skin, erythema, edema, and excoriations, and to track the effects of melittin. BV was injected peritoneally and demonstrated therapeutic effects by inhibiting mast cell infiltration, and lowering filagrine levels and the secretion of chemokines and inflammatory cytokines related to atopic dermatitis. The study concluded that melittin exhibits anti-inflammatory activity and may be included in various formulations for treating atopic dermatitis [76].

Apamine, an important component of BV, was studied by a group of researchers who discovered its anti-inflammatory effect on TNF-α- and IFN-γ-induced response in human keratinocytes. The presence of BV apamine inhibited the activation of transcription factors JAK/STAT and NF-kB, factors correlated with inflammatory cytokines in human keratinocytes treated with TNF-α and IFN-γ, all of which demonstrated a possible use in the treatment of atopic dermatitis [77]. 

Gu et al. studied the therapeutic effects of BV on experimental atopic dermatitis, using mouse models with ovalbumin-induced skin lesions (OVAs), and tracked lesion evolution and the mechanism of action of venom administered via intraperitoneal inoculation. A histological analysis of dorsal skin thickness was performed, which showed the inhibition of inflammatory cytokines though a decrease in the secretion of immunoglobulin E, thymic stromal lymphopoietin, and TNF-α. The treatment also suppressed mast cell and eosinophil infiltration into the lesion. The results of the study indicated that the use of BV may be an alternative to the treatment of atopic dermatitis, due to its proven anti-inflammatory effect [78].

A group of researchers investigated BV’s role in regulating the complement system. Atopic lesions were induced in mouse models using 1-chlorine-2,4-dinitrobenzene (DNCB) and the subcutaneous administration of BV completely resolved symptoms. A complement-dependent cytotoxicity test and bacteria kill test demonstrated that venom inactivated the complement system by inducing CD55, a complement formation inhibitor in THP-1 cells, resulting in a decrease in the serum levels of C3 convertase (C3C) and membrane attack complex (MAC). The study concluded that BV can be successfully used to treat atopic dermatitis [79].

An important symptom of atopic dermatitis is itch, which causes the desire to scratch. When excessive, this can aggravate the disease. A study was conducted on the anti-itch effect of BV in a mouse scratching behavior model induced by the 48/80 compound. The intraperitoneal administration of BV attenuated scratching behavior proportional to its vascular permeability effects, and suppressed mast cell degranulation and proinflammatory cytokine production in skin tissues treated with the 48/80 compound, improving symptoms related to atopic dermatitis [80].

Phospholipase A2, another important component of BV, was studied by Jung et al., who tracked its topical application effects on mice with atopic dermatitis induced by *Dermatophagoides farinae* extract (DFE). Epidermal thickness, the infiltration of immune cells, serum immunoglobulin, and cytokines were measured, with results showing a decrease in ear thickness, a significant reduction in serum cytokines IgE, Th1 (TNF-α, IL-6, and IFN-γ) and Th2 (IL-4 and IL-13), and the inhibition of mast cell infiltration into the ear. All these aspects suggest that the topical application of phospholipase A2 from BV may be a possible method of combating symptoms of atopic dermatitis [81,82].

A clinical trial demonstrating the effectiveness of BV on human skin was conducted by You et al. It was a double-blind, randomized, controlled, multicenter study involving 136 patients diagnosed with atopic dermatitis. Subjects were randomly assigned to different groups and given either an emollient containing BV and silk protein or a venom-free moisturizer for topical administration twice daily for 4 weeks. Severity index and eczema area score (EASI), transepidermal water loss, and visual analog scale (VAS) itch score were evaluated at the first visit, and then every 2 and 4 weeks. The results showed that subjects using the BV-containing emollient had a lower EASI score and VAS compared to subjects who applied venom-free emollient; there were no differences in the incidence of adverse reactions, suggesting that this is a possible effective and safe treatment option for atopic dermatitis [83].

#### 5.1.2. *Ficus carica* in Atopic Dermatitis

To find a natural treatment alternative for atopic dermatitis in children, a group of researchers investigated the effect of aqueous dried fruit extract of *Ficus carica* L. in mild to moderate atopic dermatitis. The clinical trial involved 45 children aged from 4 months to 14 years with mild to moderate atopic dermatitis (SCORAD < 50), who were randomly assigned, double-blind, to three treatment groups to participate in a randomized, double-blind, placebo-controlled clinical trial. The effect of an aqueous extract of dried edible fig fruit on the severity of atopic dermatitis, as measured using the atopic dermatitis (SCORAD), compared to 1.0% hydrocortisone as a routine AD treatment and base cream as a placebo, was determined. Patients were instructed to apply their assigned creams twice daily for two weeks. It was found that the placebo did not improve symptoms, and fig fruit extract significantly reduced the SCORAD index, intensity scores, and itching compared to hydrocortisone 1.0%, conferring superior safety, efficacy, and tolerability, which may suggest its use as a potential therapeutic agent for atopic dermatitis in children [84].

#### 5.1.3. Geranium Essential Oil in Dermatitis

GEO can be used in dermatitis, leading to a decrease in itching and erythema, and an acceleration of healing of tissues affected by dermatitis [85]. A group of researchers, using mice, conducted an in vivo evaluation of the potential anti-inflammatory effect of GEO when applied topically to a croton-oil-induced ear edema. A dose-dependent reduction in ear edema by GEO was observed (73% inhibition occurred at 200 μL/kg and 88% inhibition at 400 μL/kg). Diclofenac sodium (40 mg/kg), used as a reference, produced an 85% inhibition of croton-oil-induced inflammation. In addition, histological analysis confirmed that GEO inhibited inflammatory responses in the skin. All these results demonstrate the potential use of GEO in treating various inflammatory skin conditions, including atopic dermatitis [86].

### 5.2. Acne

#### 5.2.1. Bee Venom in Acne

Han et al. conducted a study evaluating the antimicrobial properties of BV against some etiological agents of acne vulgaris (propionibacterium acnes, clindamycin-resistant Propionibacterium acnes, Staphylococcus epidermidis, and Streptococcus pyrogenes). The production of inflammatory cytokines (IL-8) and tumor necrosis (TNF-α) was examined in THP-1 cells. BV was found to reduce cytotoxicity to 10 μg/mL in human keratinocytes and epidermal monocytes and reduce Propionibacterium acnes (*P. acnes*), as well as inducing the secretion of IL-8 and TNF-α into THP-1 cells, demonstrating that it can act effectively in the treatment of acne vulgaris through its antimicrobial and anti-inflammatory effects [87]. 

Melittin was studied by Lee et al. for its possible therapeutic action on inflammatory cytokine production in heat-destroyed *P. acnes*-treated keratinocytes. Treatment with melittin decreased TNF-α and IL-1β expression by regulating the NF-κB and MAPK pathways in keratinocytes. The anti-inflammatory effects of melittin were studied by the same team of researchers via the intradermal injection of *P. acnes* into the ears of mice to cause inflammation, before treating the right ears with different concentrations of melittin (1, 10 and 100 μg) mixed with 0.05 g of petroleum jelly. The results showed that melittin decreased the expression of proinflammatory cytokines regulated by transcription factors such as NF-kB and AP-1 in acne lesions, and the 100 μg concentration of melittin resulted in a 1.3-fold reduction in ear thickness compared to ears injected with *P. acnes* alone. The study authors concluded that melittin may be useful for acne care [88]. 

It has been found that, in this skin condition, the main factor producing inflammation is *P. acnes*, so researchers have particularly looked at BV’s inhibitory effects on inflammation produced by this bacterium [89,90]. An et al. injected *P. acnes* intradermally into the ears of mouse models (six groups); then, BV (1, 10, and 100 μg, mixed with 0.05 g petroleum jelly) was applied to the surface of the skin of the right ear to study the therapeutic effects. The venom was shown to significantly decrease the expression levels of tumor necrosis factor (TNF)-α and interleukin (IL)-1β and inhibit proinflammatory cytokines by modulating TLR2-mediated NF-κB and AP-1 signaling in inflamed skin tissue. All these results suggest that BV can be used to treat acne [91].

Purified bee venom (PBV) was used by Han et al. in a randomized double-blind control study on a total of 12 subjects with acne vulgaris. The cutaneous bacterium *P. acnes* was incubated with PBV at different concentrations, bacterial growth was tracked using the colony-forming unit test, and the mechanism used by venom to destroy the bacterium was examined via electron microscopy. The subjects used either venom-containing or venom-free care products for two weeks, with the results showing that patients using PBV had a 57.5% decrease in adenosine triphosphate levels compared to others, who had only a 4.7% decrease. A difference was observed between grading levels based on the number of inflammatory and non-inflammatory lesions in favor of the venom group compared to controls, and, in addition, PBV was reported to exhibit concentration-dependent antimicrobial activity. All these aspects conclude that BV can be successfully used in the formulation of acne treatment products [92].

In a prospective, non-comparative study conducted by Han et al., the effect of a purified BV serum was determined using 30 volunteers with mild to moderate acne vulgaris. The serum was applied twice daily for 6 weeks to the affected areas. A clinical evaluation of lesions was performed at weeks 0, 3, and 6, with an average percentage improvement in the degree of acne of 52.3% being observed at 6 weeks. No side effects occurred, and the subjects showed a significant improvement in comedones, papules, pustules, and nodules at the end of the study, thereby demonstrating the effectiveness of the topical administration of purified BV serum for acne vulgaris care [93].

Given that the use of microcapsules or liposomes laden with different active substances to treat acne had a beneficial role over a long period of time due to the retarding effect obtained by encapsulating the active principle, this is an opportunity to investigate whether encapsulated BV would have the same action over a longer period [94].

#### 5.2.2. *Ficus carica* in Acne

Recently, a group of researchers set out to formulate and evaluate an acne cream made from fig leaf extract. Methods included the formulation and evaluation of anti-acne cream, an analysis of its antibacterial activity against *P. acnes* and *Staphylococcus epidermidis* (*S. epidermidis*), and irritation test, and a preference test. Oil-in-water creams were formulated with varying extract concentrations (1, 2, and 3%) and were observed for 8 weeks. The creams had a uniform and smooth consistency, with a more intense fragrance and color as the concentration of fig leaf extract increased. The homogeneity test confirmed the uniform distribution of phases, maintaining the stability of the cream. Its antibacterial efficacy against *P. acnes* and *S. epidermidis* was influenced by the concentration of the extract. The preferred formula, due to its texture and fragrance, was the one containing the extract in 3% concentration. No cream produced discomfort, swelling, or redness, indicating that both vehicle substances and fig leaf extract are safe for topical use [95].

#### 5.2.3. Geranium Essential Oil in Acne

Due to the high concentrations of linalool, citronellol, and geraniol present in its composition, GEO contributes to regulating the moisture balance of the skin, improvements in skin blood circulation, and the regeneration of skin cells, which are very important to mitigate the effects of acne [73]. A group of researchers evaluated the sebostatic activity of *Juniperus communis* fruit oil and *Pelargonium graveolens* oil compared to niacinamide. Five tonics with varying concentrations of oil were prepared and applied to the skin of six people in the study. Measurements of sebum production on the forehead, cheek, and forearm were performed using a Sebumeter^®^ SM 815 (Courage & Khazaka®, Köln, Germany), and the results showed the superior efficiency of the tonic containing 0.25% *Pelargonium graveolens* oil in reducing sebum production [15].

### 5.3. Psoriasis

Current therapies are not sufficient to cure the disease, so various studies are being conducted, with some of the researched products including BV and FC.

#### 5.3.1. Bee Venom in Psoriasis

Hegazi et al. evaluated BV and propolis as new therapeutic modalities for localized plaque psoriasis. The study involved 48 patients, randomized to four groups receiving different treatments. The first group received intradermal BV, the second group received propolis ointment, the third group received a propolis capsule, and the last group was treated with intradermal venom group propolis capsules and ointment. Treatment response was assessed by calculating the Psoriasis Area and Severity Index (PASI) score and measuring serum interleukin-1β (IL-1β) before and after 3 months of treatment. It was observed that both PASI score and serum IL-1β levels showed a significant decrease in patients who received BV intradermally compared to other treatment groups, and the side effects were minimal, demonstrating that it can be successfully used to treat this condition [96].

A randomized double-blind clinical trial was conducted by Eltaher et al., who used BV as a possible curative agent to treat recalcitrant localized plaque psoriasis (PPR). The study involved 50 patients with PPR; 25 received BV injected into lesions weekly for 12 weeks, and the other 25 received a placebo. The level of tumor necrosis factor alpha (TNF-α) was measured before the study and at week 12, and a significant decrease in TNF-α was observed in the venom group compared to the placebo group. No major adverse effects were reported, and a complete response occurred for 92% of patients with PPR receiving BV. At the same time, after 6 months, no relapse was observed in these patients, which demonstrates the effectiveness and the possibility of using this product as a therapeutic agent to treat this condition [97]. 

#### 5.3.2. *Ficus carica* in Psoriazis

FC is known for its psoralen content, with beneficial pharmaceutical activities on the skin [98]. Psoralen plus ultraviolet A photochemotherapy (PUVA) is an FDA-approved treatment that combines the administration of psoralenes with exposure to ultraviolet A (UVA) radiation. PUVA is used to treat a variety of skin diseases, including psoriasis [99].

Lee et al. analyzed the anti-psoriasis effect of FC fruit extract, both in cell cultures (in vitro) and in mice (in vivo). The results showed that the extract reduced nitric oxide production, and iNOS and COX-2 expression in inflammatory-activated cells significantly influenced the JAK-STAT signaling pathway associated with psoriasis and reduced the release of β-hexosaminidase. In mice, extract treatment decreased skin thickness and PASI score, and decreased epidermal dermis thickness, in addition to reducing STAT3 phosphorylation, all of which demonstrate that FC fruit extract may be a potential therapeutic agent for psoriasis [100].

### 5.4. Wound Healing

#### 5.4.1. Bee Venom in Wound Treatment

BV, due to its components’ multiple proven effects, including anti-inflammatory, antioxidant, antimicrobial, and analgesic effects, has aroused the interest of many researchers for its possible use in healing wounds at different stages, especially the wounds of diabetic patients, which are often causes of morbidity and mortality [101].

To study the effect of BV in wound healing, Han et al. used mouse models with wounds in the dorsal area, which were divided into three groups (control, petroleum jelly, and venom). Their relative sizes were measured, and histological wound tests were performed after 3, 5, and 7 days. The treatments were applied to the gauze covering the wounds, and the results showed that the wounds of the BV-treated group closed considerably faster compared to the other two groups. Immunohistochemical staining indicated that BV reduced fibronectin levels, transforming growth factor β1, and vascular endothelial growth factor, but increased collagen-I expression. The reported aspects concluded that venom can be used topically for wound care [102].

The molecular mechanisms underlying BV treatment for healing wounds caused by diabetes have been studied by Hozzein et al. Three experimental groups were studied: nondiabetic control mice, vehicle diabetic mice, and BV-treated diabetic mice. The results showed that BV treatment improved wound closure in diabetic mice by increasing collagen and β-defensin-2 expression, restoring angiopoietin-1 (Ang-1) and nuclear-factor E2-bound factor 2 (Nrf2) levels, and improving downstream receptor tyrosine-protein kinase (Tie-2) signaling. At the same time, it was observed that the administration of BV to mice with diabetes restored the actions of antioxidant enzymes, damaged tissues and chemokine levels, and subsequently saved macrophages from mitochondrial apoptosis. All these results indicate that BV could be a therapeutic agent for healing wounds caused by diabetes [103]. 

Amin et al. developed freeze–thaw method dressings with different concentrations of polyvinyl alcohol, chitosan, and BV to study the possibility of wound healing in diabetic rats. It was concluded that BV-laden hydrogel made from 10% polyvinyl alcohol, 0.6% chitosan, and 4% venom was more flexible and elastic than other formulations, led to faster wound healing in diabetic rats compared to the control, and also demonstrated an anti-inflammatory effect comparable to the standard anti-inflammatory product (diclofenac gel) [104].

The impact of BV on wound healing in type I diabetic mouse models was also investigated by Badr et al., who found that venom treatment improved wound healing in diabetic mice by restoring levels of inflammatory cytokines, free radicals, TGF-β, and VEGF, and increasing collagen production. BV has also been reported to accelerate the healing process in experimental animals by affecting the activity of caspase-3, caspase-8, and caspase-9 [105].

#### 5.4.2. *Ficus carica* in Wound Treatment

Current studies have shown significant efficacy in the treatment of diabetes lesions by applying dressings containing polymers, nanoparticles, and various plant compounds [106]. A group of researchers created a biodegradable matrix from a material with an affinity for hydrophilic and hydrophobic substances, poly xylitol derivative, and polyhydroxy butyrate enriched with FC extracts to study the regeneration of tissues affected by diabetic wounds. An amphiphilic polymeric skeleton loaded with *Ficus carica* extract (FFE) of poly (xylitol-g-adipate-co-glutamide) (PXAG)-polyhydroxybutyrate (PHB) was made. The PXAG copolymer was prepared via the condensation method, and the PXAG-PHB, PXAG-PHB/FFE polymer scaffolding was created using the ultrasonic process and magnetic stirring processes. The influence of the dressing on wounds was determined using the in vitro scratch wound test in diabetic wound cell models, and it was observed that those treated with PXAG-PHB/FFE showed complete healing within 72 h compared to control cells, demonstrating that the tested treatment has the potential to function properly in diabetic wound cell models. The biomaterial PXAG-PHB/FFE had thermal stability, did not generate adverse reactions, reacted with biological tissues stimulating favorable reactions from the body, and was shown to have strong antimicrobial activity on pathogens *Escherichia coli* and *Staphylococcus aureus*. The originality of the compound effectively contributes to improved antioxidant, and anti-inflammatory activities and promotes cell proliferation, leading to the formation of new blood vessels by stimulating the multiplication of endothelial cells. All of these factors are closely related to tissue regeneration in diabetic wounds, which means this treatment has significant potential in terms of its applicability to tissue regeneration for the treatment of diabetic wounds [107].

#### 5.4.3. Geranium Essential Oil in Wound Treatment

Research conducted in Poland aimed to determine the antibacterial activity of *Geranium* oil against five genera of Gram-negative clinical isolates from patients with hard-to-treat wound infections. The study included 63 patients, 38 men and 25 women, with an average age of 46–58 years, all with wounds arising during diabetes or unhealed wounds after burns, and samples of Gram-negative clinical strains were isolated from patients’ swabs. GEO, tested with a composition mainly comprising citronellol (26.7%) and geraniol (13.4%), inhibited the growth of all Gram-negative clinical strains of *Escherichia coli*, *Citrobacter freundii*, *Enterobacter sakazakii*, *Enterobacter cloacae*, *Proteus mirabilis*, and *Pseudomonas aeruginosa* at concentrations ranging from 3.0 μL/mL to 10.5 μL/mL. The results suggest that it can be used as a therapeutic agent to treat wounds [108].

### 5.5. Alopecia

#### 5.5.1. Bee Venom in Alopecia

BV is a promising candidate for alopecia treatment due to its anti-inflammatory, immunomodulatory, and circulation-enhancing properties [109,110]. Park et al. investigated the preventive effect of BV for alopecia by applying it at different concentrations (0.001, 0.005, and 0.01%) to the dorsal skin of female C57BL/6 mice for 19 days, using minoxidil 2% as a positive control. The factors responsible for hair growth were studied by quantitative real-time PCR and Western blot analysis using mouse skin and human dermal papilla cells (hDPCs). BV did not cause edema, irritation, or cytotoxicity at the concentrations used and it was observed that, in female C57BL/6 mice, the local administration of a BV concentration of 0.01% improved hair growth. The results showed that BV improves hair follicle development by reducing 5α-reductase expression, and stimulates the expression of growth factors such as vascular endothelial growth factor (VEGF), insulin-like growth factor receptor 1 (IGF-1R), fibroblast growth factor 7 (FGF7), and fibroblast growth factor 2 (FGF2). It also hinders the catagen process and improves the proliferation of human dermal papilla cells in a dose-dependent manner compared to the control. The specialists concluded that BV is a potential inhibitor of 5α-reductase and a promoter of hair growth [111].

The results of a recent study conducted by Kim et al. suggested that BV can be used as a fat-derived stem cell (AUC) preconditioning agent for hair regrowth. AUCs treated with BV were injected subcutaneously into mice, with an acceleration of change from telogen to anagen, and, after 14 days, hair weight increased. The quantitative polymerase chain reaction (qPCR) showed that BV influenced the expression of growth factors, transcription factors, chemokines, and enzymes, and the Boyden chamber experiment and scratch test showed an increased regulation of cell migration by the venom. These results demonstrate its potential use in the treatment of hair loss [112].

#### 5.5.2. *Ficus carica* in Alopecia

Recently, very small particles, of the order of nanometers, with a high degree of biodegradation, that are based on polymers have become of great interest in the pharmaceutical and cosmetic fields [113]. Microscopic particles based on poly-γ-glutamic acid (γ-PGA) are promising drug delivery vectors due to their ability to deliver drugs in a controlled manner while being safe and biologically compatible [114]. To increase the transfer of hair growth agents to the scalp, a group of researchers applied a delivery technology that encapsulates effective hair growth products into nanoparticles. For this purpose, 4HGF was created, a plant blend of *Phellinus linteus* grown on germinated brown rice, *Cordyceps militaris* grown on germinated soybeans, *Polygonum multiflorum*, FC, and *Coconut nucifera* oil. 4HGF was encapsulated in PGA/chitosan nanoparticles (PGA-4HGF) and the effect of hair growth was investigated via in vitro and in vivo studies [115]. In vivo tests were performed on C57BL/6N mice at the telogen stage, when the dorsal skin has a pink tinge; in the anagen phase, it becomes more pigmented. The PGA-4HGF-treated groups showed more pigmentation than the control groups, suggesting an improvement in active hair growth. The PGA-4HGF treatment group showed a significant increase in the length of regenerated hair (3.89 ± 1.04 mm) compared to the control group (1.89 ± 0.58 mm) [115]. PGA-4HGF was also found to activate the β-catenin pathway, promoting the G1/S transition by regulating cyclinD1 and CDK4 proteins, as well as increasing keratin type II proteins and melanin, contributing to sustainable hair growth. All these results suggest that the use of PGA nanocapsules for the release of 4HGF could be a viable therapeutic option for addressing hair growth issues [115].

### 5.6. Wrinkles

The desire to prevent and alleviate facial wrinkles has led to the study and formulation of many products to improve the appearance of the skin, with many of them using natural ingredients, including BV, FC, and GEO.

#### 5.6.1. Bee Venom for Wrinkles

A group of researchers conducted a study evaluating skin sensitization to BV in guinea pigs and rats using the Buehler test. The skin response, assessed by erythema and edema at the challenge sites, and the sensitization rate in the BV-sensitization rat group were much lower compared to those in the positive control group, demonstrating minor venom-sensitization potential. These results showed that BV could be safely used in formulations and topical human use [116].

The first clinical study to determine the effect of BV cosmetics on human facial wrinkles was conducted by Han et al. This study evaluated the beneficial effects of a facial serum containing a concentration of 0.006% BV, with 4 mL being applied to the face twice daily for 12 weeks by 22 volunteers, comprising South Korean women aged 30–49 years. To observe changes in skin wrinkles, visual evaluations were performed by the dermatologist, photographs were analyzed, and an image analysis was conducted of replicas. It was concluded that treatment with serum containing BV produced a clinical improvement in facial wrinkles by decreasing the total area, number, and average depth of wrinkles without producing side effects; therefore, it can be successfully used to treat wrinkles [117].

#### 5.6.2. *Ficus carica* for Wrinkles

The antioxidant, anti-collagenase (in vitro), and anti-wrinkle (in vivo) effects of a combined formulation containing *Ginkgo biloba, Punica granatum*, FC, and *Morus alba* fruit extract was studied and demonstrated by a group of researchers. This study was a randomized, open-label, single-blind, placebo-controlled, observer-blind study involving 21 women aged 45–65 years, who were treated with 2% locally formulated fruit extract on one side of the face and a placebo on the other side of the face, twice daily, for 56 days. Treatment for 28 days showed no significant differences from the inactive substance, but after 56 days, the depth, length, and area of wrinkles decreased significantly compared to the inactive topical application [118]. The antioxidant evaluation was based on free radical capture activity (1,1-diphenyl-2-picrilhydrazil, H_2_O_2_ and O_2_^−^). The ethanolic extracts obtained from four types of fruits demonstrated strong antioxidant activity. The IC50 values for free radical capture and hydrogen peroxidation inhibition showed a comparable or even superior efficacy to the standard compounds used in in vitro testing. The extract also showed oxygen radical inhibition in a dose-dependent manner. The anti-collagenase activity was based on an in vitro reduction in collagenase enzyme, with the combined fruit extracts inhibiting these enzymes at varying levels depending on the dose. At a concentration of 5 μg/mL, the extract inhibited the enzyme by 67.45%, while lower concentrations resulted in inhibitions of 12.03%, 32.90%, and 55.61% [118]. The results proved that the combined formula of fruit extracts (*Punica granatum,* FC, *Morus alba*, and *Ginkgo biloba*) has excellent antioxidant and anti-collagenase activity, as well as a significant anti-wrinkle effect on human skin [118].

Khan et al. investigated the effects of a cream containing *Ficus carica* L. fruit extract on various skin characteristics, such as skin melanin, erythema, moisture content, trans-epidermal water loss, and sebum. A formulation with 4% concentrated FC fruit extract and an extract-free base were developed, both of which were used twice daily for 8 weeks on the cheeks of 11 Asian male volunteers, aged 20–35 years, with no known dermatological pathologies. Non-invasive determinations were made using bioengineering techniques, using laboratory equipment such as the Mexameter reflection spectrophotometer (for melanin and erythema analyses), Tewameter MPA 5 and MPA 5 chronometer (for an analysis of water loss from the dermis and percentage of water of the corneum), and MPA 5 sebometer (for an analysis of sebum secretion) [119]. To assess the potential for irritation, patches were applied to both of the volunteers’ forearms, each with a base or formulation. After 48 h, a dermatologist assessed the presence of skin irritation. It was found that, after applying the base, small fluctuations in skin melanin levels were observed, while the use of the formulation indicated a steady reduction in melanin content throughout the entire study due to the presence of tyrosinase inhibitory phytosubstances known for their pigmentation-reducing properties [119]. From these determinations, it was observed that the presence of antioxidant substances and vitamin C in fig extract contributes to reductions in trans-epidermal water loss and stimulates collagen production. Additionally, the amount of sebum was shown to be significantly decreased, demonstrating that a stable topical cream containing FC fruit extract can have anti-wrinkle or anti-acne effects. However, further targeted studies are needed [119].

#### 5.6.3. Geranium Essential Oil for Wrinkles

A study conducted by Lohani et al. aimed to evaluate the aging potential of a cream containing GEO/ethanolic lipid vesicles (ELVs) trapped in *Calendula* essential oil. The encapsulation of *Geranium*/*Calendula* essential oils in ethanolic lipid vesicles was carried out to prevent their evaporation and to increase their availability and efficacy in cosmetic products. Two types of cream formulations were prepared, one conventional and one with ELV tissue, and tested for their homogeneity, viscosity, pH, gradability, sun protection factor, antioxidant capacity, collagenase, and elastase inhibition ability, with the results demonstrating that ELVs were able to retain the effectiveness of essential oils and have the potential to deliver active substances deeper into the skin. The researchers concluded that, due to the ethanolic lipid vesicles, cream composition, and *Geranium* and *Calendula* essential oils, a cumulative protective effect was produced to combat skin aging [120].

### 5.7. Melanoma

#### Bee Venom in Melanoma

No human studies have been conducted on the use of BV to treat melanoma, but in vitro research has yielded encouraging results. A study conducted by Lim et al. investigated the inhibitory effect and mechanism of action of BV and melittin against melanoma cells, including B16F10, A375SM, and SK-MEL-28. BV and melittin have antimelanogenic activity in the state of stimulation of α-MSH, as they strongly suppress several oncogenic processes, including growth, clonogenicity, migration, invasion, and melanogenesis, in malignant melanoma cells. Antimelanoma activity is related to the suppressive effect on PI3K/AKT/mTOR and MAPK signaling pathways. Researchers reported that melittin inhibits melanoma cell growth by inducing caspase-dependent apoptosis and had stronger anticancer effects than BV, demonstrating that this can be used in the formulation of products to treat melanoma [121]. 

BV has been reported to induce calcium-dependent and caspase-independent apoptotic cell destruction in A2058 human melanoma cells. BV-induced apoptosis is accompanied by the generation of reactive oxygen species and the deterioration of mitochondrial membrane potential transition. The treatment of A2058 cells with melittin resulted in good cell-destroying effects, suggesting that this is the component of BV that is responsible for its antiproliferative action, and that it may be a potential melanoma treatment agent [122]. 

The anticancer effect of melittin was studied by Soman et al. to treat mouse melanoma B16F10. They used melittin-laden perfluorocarbon nanoparticles, which were intravenously provided to mice, resulting in a tumor shrinkage of about 87 percent compared to the controls. The histological analysis showed a decrease in the number of proliferating cells and areas of necrosis, but the lack of toxic effects was also remarkable. Based on these results, the researchers demonstrated that nanoscale synthetic vehicles can successfully use melittin, through molecular targeting, for the treatment of melanoma [123].

### 5.8. Melasma

#### *Ficus carica* in Melasma

To find suitable natural alternatives for the treatment of melasma, a group of researchers studied the effectiveness of a topical formulation called Tila-e-Kalf (a homogeneous paste containing *Lens culinaris* powder, *Prunus amygdalus,* and FC decoction) from the Unani Pharmacopoeia in a randomized controlled clinical trial. Sixty-five patients diagnosed with melasma, including both women and men over 18 years of age, were supervised and followed by a computerized randomization chart. Patients were divided into the tested group (who received Tila-e-Kalf) and control group (who received hydroquinone 4% cream). The test group included 32 patients and the control group included 33 patients; only 55 patients completed the test. Testing lasted 8 weeks, with one additional application day and night, for both groups. During the initial evaluation, the patient’s medical history was collected, a facial examination was performed, and melasma severity index (MASI) score, dermatological quality of life index (DLQI) score, and physician global assessment (PGA) score were recorded. These MASI, DLQI, and PGA scores were reviewed at each follow-up meeting at two-week intervals. Color photographs of the face were captured at the beginning and end of treatment according to the established protocol. In this study, both hydroquinone 4% and Tila-e-Kalf showed similar efficacy for several indicators. Adverse reactions were reported by patients in the control group due to the use of hydroquinone in the 4% topical preparation, while the tested drug only caused mild itching in one patient. Conclusions regarding the safety and efficacy of this treatment suggest that Tila-e-Kalf, which also contains the FC decoction, can be more successful in the long-term compared to hydroquinone, a conventional treatment that is more commonly prescribed in the management of melasma, as well leading to fewer adverse reactions [124].

### 5.9. Morphea

#### Bee Venom in Morphea

Hwang et al. conducted a clinical trial and obtained very promising results regarding the use of BV acupuncture for circumscribed morphea in a 64-year-old systemic sclerosis patient who was initially evaluated for venom compatibility. The patient had white circular areas with a diameter of 1 and 3 cm and intense itching in the right lateral iliac ridge. The treatment was carried out over four weeks; BV was administered twice via subcutaneous acupuncture in the first week, then once a week for another 3 weeks, and the only adverse effect was local pruritus after the sting. Gradual reductions in itching and a significant improvement in skin condition were observed, and a follow-up evaluation after three months confirmed the appearance of normal-appearing skin. The results of this study demonstrated that BV can be successfully used in the treatment of this condition [125].

### 5.10. Vitiligo

Scientists are interested in discovering alternatives, preferably natural [126], for the treatment of this condition, and BV belongs to the category of investigated substances.

#### Bee Venom in Vitiligo

Jeon et al. studied BV’s action on proliferation, melanogenesis, dendricity, migration into normal human melanocytes, and signal transduction. The results showed that BV induced melanogenesis by increasing tyrosinase expression, increased melanocyte count by activating PKA, ERK, and PI3K/Akt, and induced dendricity and melanocyte migration by activating PLA(2). All these actions demonstrate that BV has the potential to cause skin repigmentation in vitiligo [127]. 

The predominant secretory phospholipase expressed by keratinocytes is secretory phospholipase group X A2 (sPLA2). Studies have proven that this stimulates the centricity and pigmentation of cutaneous melanocytes through a mechanism dependent on lysophosphatidylcholine, and that it is a good mediator of post-inflammatory or UV-induced pigmentation [128]. A group of researchers used organ-grown guinea pig skins to study melanogenic responses to exogenous stimulation. An increase in melanogenic activity was observed following UV irradiation. Phospholipases, arachidonic acid, interleukin-1 alpha, and melanocyte-stimulating hormone-boosted melanogenesis, especially PLA2, showed a superior stimulating capacity, suggesting their potential use in epidermal hyperpigmentation [129]. 

### 5.11. Fungal Infections

#### 5.11.1. Bee Venom in Fungal Infections

Following the results of various other studies, BV has been proven to have remarkable antifungal effects against *Trichophyton mentagrophytes* and *Trichophyton rubrum*, *Candida albicans*, and *Malassezia furfur* [51,130,131]. Park et al. conducted an in vitro study to evaluate the antifungal effects of bee venom components in order to identify a possible component/active substance that prevents the growth and spread of *T. rubrum*. The results showed that BV, in its entire form, had better effects than venom in separate component forms and can be successfully used in the formulation of potential antifungal therapies [51].

#### 5.11.2. Geranium Essential Oil in Fungal Infections

The antifungal properties of GEO inhibit the growth and development of dermatophyte fungi or *Candida albicans* fungi. Thus, this type of oil is very beneficial and useful in treating onychomycosis and mycosis in various broad-spectrum treatments, in both dermatology and oral pathology [132,133]. 

The results of all studies show that BV, FC, and GEO can be effective in skin care and treatment; moreover, we observed that, in some cases, they can be used in potential therapies for the same condition (Table 1, Table 2 and Table 3).

## 6. Safety Profile and Challenges in BV, FC, and GEO Use

As a result of the research, BV is considered a therapeutic alternative in a large number of conditions, but data on its safety are still incomplete. Allergic reactions remain the main challenges facing the approval and habitual application of BV, and hypersensitivity to the venom can be fatal if it evolves into an intense systemic allergy [134]. The adverse effects of BV range from mild skin reactions that recede after a few days to severe or fatal anaphylactic responses. It has been reported that, for sensitive individuals, the administration of 100 micrograms/mL of BV may cause pain, dyspnoea, nausea, unconsciousness, or paralysis of limbs, and doses ranging from 2.8 to 3.5 mg/kg of the body weight may be lethal (LD50). The severity varies depending on the venom concentration, patient weight, immunity, prior sensitivity, and age [135]. Furthermore, a case report showed that anaphylaxis can also occur in patients who had no adverse reactions after previous BV therapy [136]. BV contains 12 main allergens, which are multiple protein allergens that possess enzymatic activity and are responsible for an allergic response. The most important are phospholipase A2, melittin, acid phosphatase, and hyaluronidase [137]. Phospholipase A2 is the most allergenic protein and is responsible for inducing immunoglobulin E (IgE), melittin induces minor allergic reactions, acid phosphatase releases histamine from sensitized human basophils, and hyaluronidase participates in the spread of apitoxin throughout the body by altering the cell membranes [135,137].

It has been demonstrated that nanoparticles can be used for sustained release because the degradation period can be set from days to years by changing the type and amount of polymer, the molecular weight of the polymer, or its structure, making it suitable for the possibility of BV delivery [138]. Poly(D, L-lactide-co-glycolide) (PLGA) has been widely studied as a carrier for drug delivery systems (DDS) proteins and peptides. It is one of the most biocompatible, biodegradable, and non-toxic materials used in the preparation of nanoparticles [139].

Park et al. conducted a BV preformulation study for the preparation of venom-loaded PLGA nanoparticles to achieve an adequate sustained release system. This required prior characterization of the physicochemical properties of BV. The venom-loaded PLGA particles (53.3% melittin) were prepared with the organic solvent dichloromethane and the ultrasonic emulsification time was two minutes. This study provided the experimental parameters needed to make venom-laden PLGA particles. Future studies could include process optimization and various tests [140].

No serious side effects were reported in humans following FC use, suggesting that its use may be considered safe [141].

GEO, when used correctly, is considered safe to use on the skin for most people, but sometimes rashes or a burning sensation may occur. It is advisable to apply GEO to the skin only after dilution with a carrier oil [74].

## 7. Conclusions

The use of various natural products to treat diseases has become increasingly popular globally, as this can avoid the sometimes severe side effects of some therapeutic chemical agents.

BV was proven to be a natural toxin with an important role in the treatment and care of the skin due to its anti-inflammatory, antimicrobial, antifungal, anticancer, antiviral, and antiaging actions.

A challenge for researchers is studying the safety of the topical application of BV, including tracking its cytotoxic and phototoxic effects.

Processing BV components through purification, modification, or nanotechnology could remove its side effects and limit its toxicity. Much research has been carried out regarding this, but efforts are still being made to introduce bee venom into the formulation of with maximum safety.

We believe that further studies are needed to better understand the action, efficacy, and safety of individual constituents of BV to prove that it can be used as a valuable therapeutic agent for various skin conditions, either when used alone or in combination with conventional medicines.

Research in the field of dermatology supports the use of FC as a beneficial natural ingredient in cosmetics and therapeutics intended for skin care. However, more studies are needed to fully understand the potential and the mechanisms of action of this shrub in the treatment of dermatological conditions, and for the development of new therapies and cosmetic formulations.

GEO, through its scientifically proven pharmacological actions, has shown that it can be successfully used, both preventively and curatively, in dermatology and oral pathologies.

A promising solution is the development of modern delivery systems for natural active principles by incorporating them into microcapsules, nanocapsules, or liposomes that allow for a sustained release, which is important in obtaining high compliance.

This review contributes to improving the knowledge of the actions of three natural products with biologically active beneficial activities in dermatology, which has a significant impact on patients’ quality of life.

## Figures and Tables

**Table 1:** Studies based on the use of BV in various skin diseases.

Nr.	Skin Diseases	Bee Venom		
		Human Studies	Anial Studies	In Vitro Studies
1.	Atopic dermatitis (DA)	Double-blind, randomized, multicentre study with q36 patients [83]	Mouse models with 1-chloro-2,4-dinitrobenzene-induced DA [75,79] Mice models with ovalbumin-induced DA [76,78] Mice models with 48/80-induced DA symptoms [80] Mice models with DA induced by *Dermatophagoides farinae* extract [82]	TNF-α/IFN-γ stimulated human keratinocytes [75] TNF-α/IFN-γ stimulated human keratinocytes [77]
2.	Acne	Double-blind, controlled study of 12 patients [92] Prospective, non-comparative study with 30 subjects [93]	Intradermal injection of *P. acnes* into mouse ears to cause inflammation [88,91]	Production of inflammatory cytokines (IL-8) and tumor necrosis (TNF-α) was examined in THP-1 cells [87] Effect of melittin on inflammatory cytokine production in heat-destroyed *P.acnes*-treated keratinocytes [88]
3.	Psoriasis	48 patients, randomized to four different treatment groups [96] Randomized double-blind study with 50 patients [97]		
4.	Wound		Mice models with dorsal wounds [102] Type I diabetic mouse models with diabetic wounds [103,105] Diabetic rat models with wounds [104]	
5.	Alopecia		Dorsal skin of female C57BL/6 mice [111] BV-treated, adipose-derived stem cells were injected subcutaneously into mice [112]	
6.	Wrinkles	Clinical study with 22 female volunteers [117]	Assessment of skin sensitization to BV in guinea pigs and rats [116]	
7.	Melanoma	There are no human studies at present	Perfluorocarbon nanoparticles loaded with melittin for mouse melanoma B16F10 [123]	B16F10, A375SM, and SKMEL28 melanoma cells [121] Human melanoma cells A2058 [122]
8.	Morphea	Clinical study; patients of 64 years; acupuncture [125]		
9.	Vitiligo			Human melanocytes [127] Guinea pig skins grown in organs [129]
10.	Fungal infection			Forty-eight plates inoculated with *Trichophyton rubrum* [51] Ten clinical isolates of *C. albicans* that were cultured from blood and vagina via the disc diffusion method [131]

**Table 2:** Studies based on the use of FC in various skin diseases.

Nr.	Skin Diseases	*Ficus* *carica*		
		Human Studies	Animal Studies	In Vitro Studies
1.	Atopic dermatitis	The randomized, placebo-controlled clinical trial involved 45 children aged from 4 months to 14 years [84]		
2.	Acne	Volunteer group for irritation test and preference test [95]		
3.	Psoriasis		Mouse model of IMQ-induced psoriasis [100]	LPS-stimulated RAW 264.7 cells [100]
4.	Wound			Human wound cells (WS1) were cultured in Eagle Environment Modificat Dulbecco [107]
5.	Alopecia		Telogen-stage C57BL/6N mouse models [115]	HaCaT cells (5 × 103 cells/well); normal human keratinocytes [115]
6.	Wrinkles	Randomized, open-label, single-blind, placebo-controlled trial with 21 women (age 45–65 years) [118] Simple, blind, and comparison study with 11 Asian men [119]		
7.	Melasma	Randomized controlled clinical trial; 65 patients diagnosed with melasma; women and men over 18 years of age [124]		

**Table 3:** Studies based on the use of GEO in various skin diseases.

Nr.	Skin Diseases	*Geranium* Essential Oil		
		Human Studies	Animal Studies	In Vitro Studies
1.	Atopic dermatitis		Croton-oil-induced ear edema in mouse models [86]	
2.	Acne	Evaluation of sebostatic activity on 3 women and 3 men [15]		
3.	Wound			Samples of Gram-negative clinical strains were isolated from swabs from serious injuries of 63 patients [108]
4.	Wrinkles		Skin irritation study in rat models [120]	Collagenase inhibition test [120]

## Data Availability

Not applicable.
